# Supplementary material for: A novel role for CSA in the regulation of nuclear envelope integrity: uncovering a non-canonical function
Source: Life Sci Alliance. 2024 Aug 29;7(11):e202402745. doi: 10.26508/lsa.202402745 (PMC11361374; doi:10.26508/lsa.202402745)
Supplement: Supplementary file 1 [file LSA-2024-02745_SdataF1_F2_F4_F7_F8_F9_FS1_FS4_FS5.pdf]

A Novel Role for CSA in the Regulation of Nuclear Envelope Integrity: Uncovering a Non-Canonical Function

Full Western Blot

Figure 1B

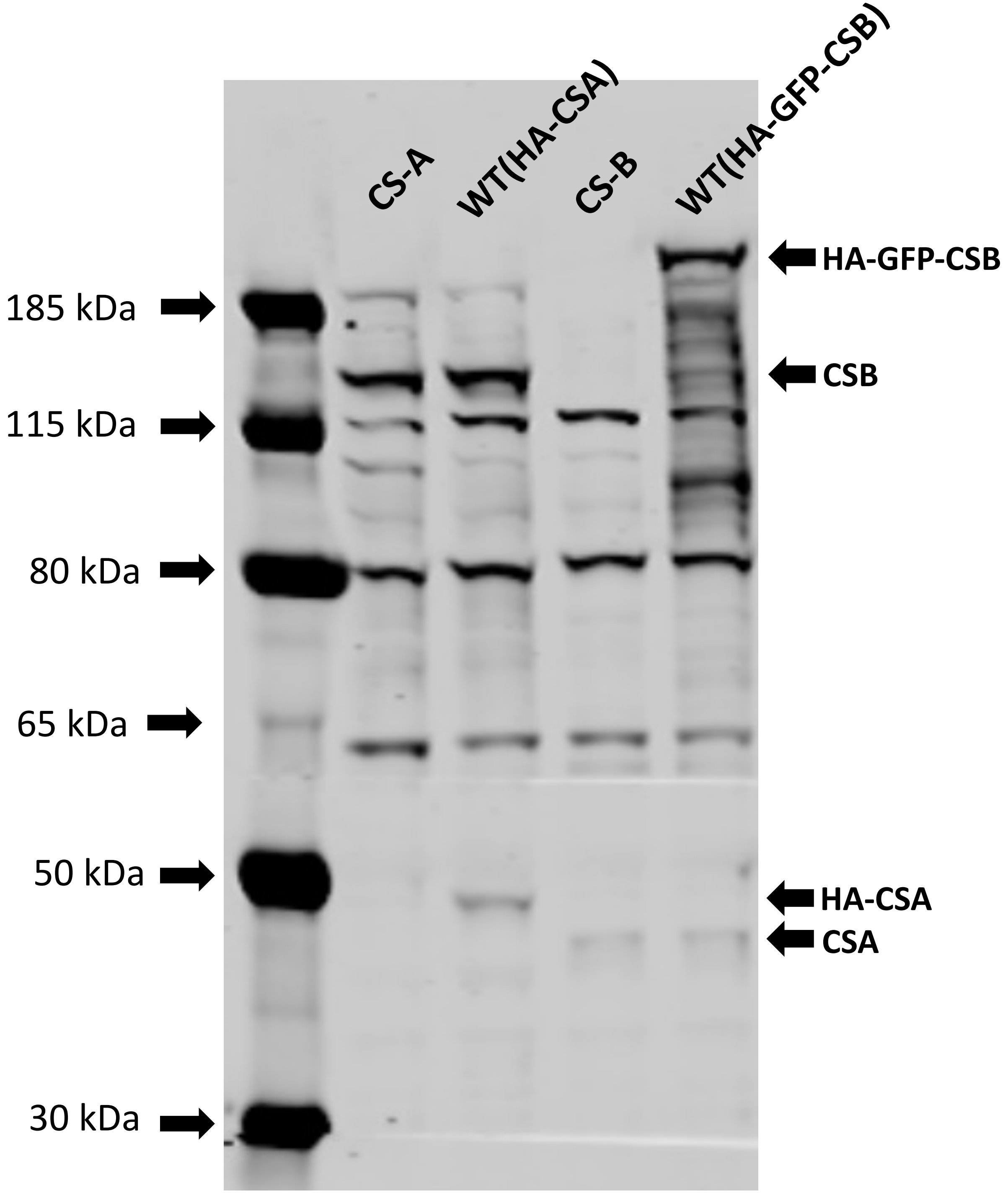
CS-A
WT(HA-CSA)
CS-B
WT(HA-GFP-CSB)
185 kDa
115 kDa
80 kDa
65 kDa
50 kDa
30 kDa
HA-GFP-CSB
CSB
HA-CSA
CSA

Figure 2A Left blot

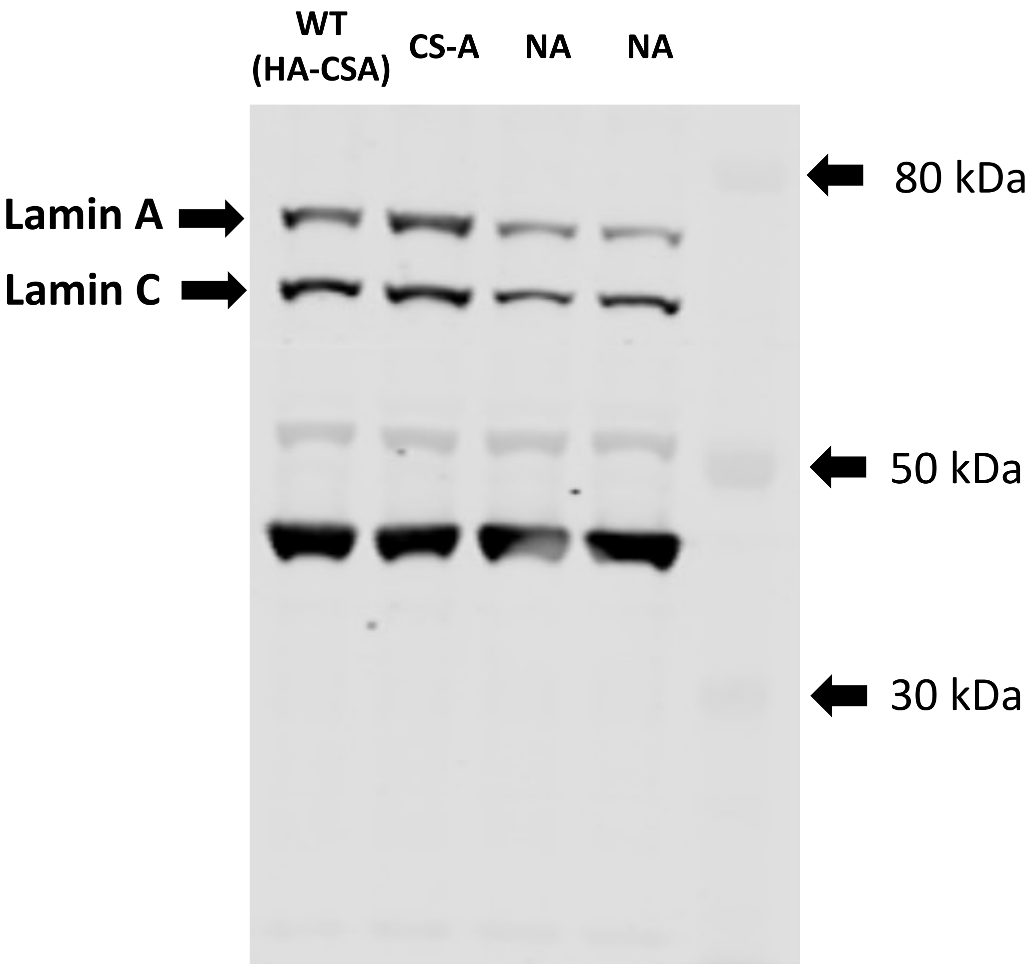

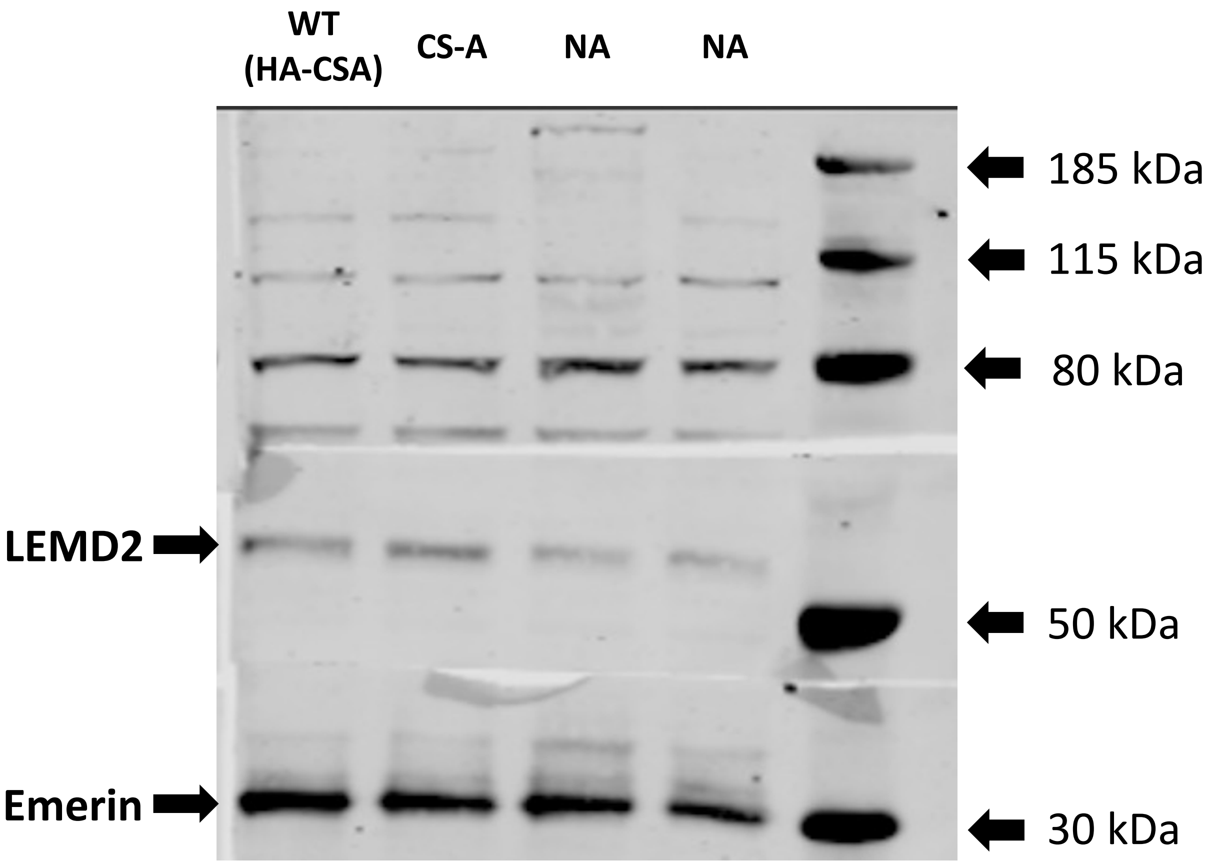

Figure 2A Left blot

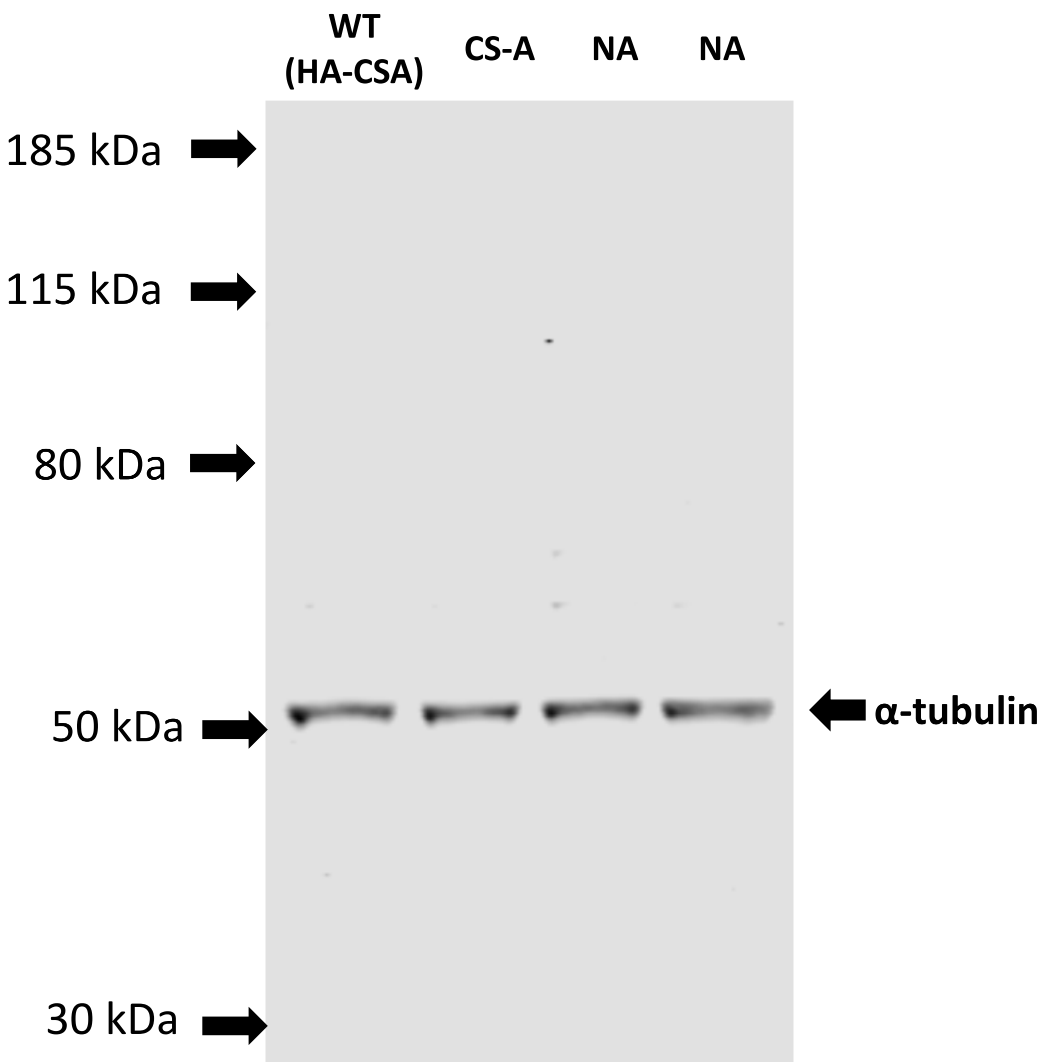

Figure 2A Right blot

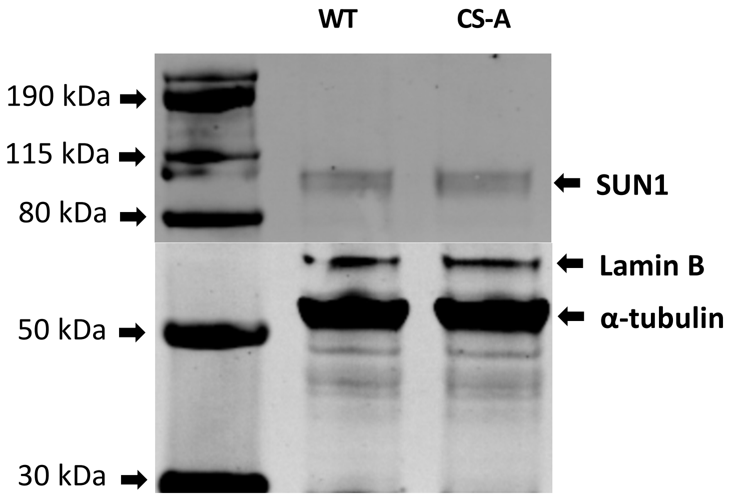

Figure 2E

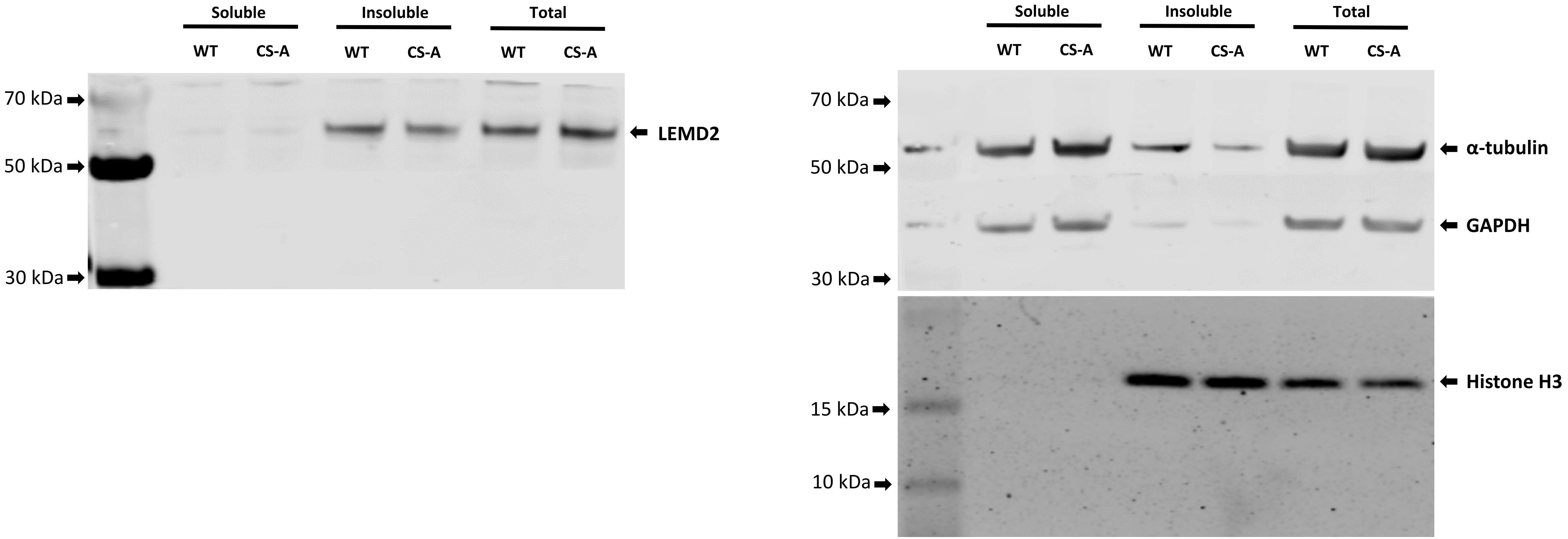
Soluble
Insoluble
Total
WT
CS-A
WT
CS-A
WT
CS-A
70 kDa
50 kDa
30 kDa
LEMD2
Soluble
Insoluble
Total
WT
CS-A
WT
CS-A
WT
CS-A
70 kDa
50 kDa
30 kDa
15 kDa
10 kDa
α-tubulin
GAPDH
Histone H3

Figure 4A

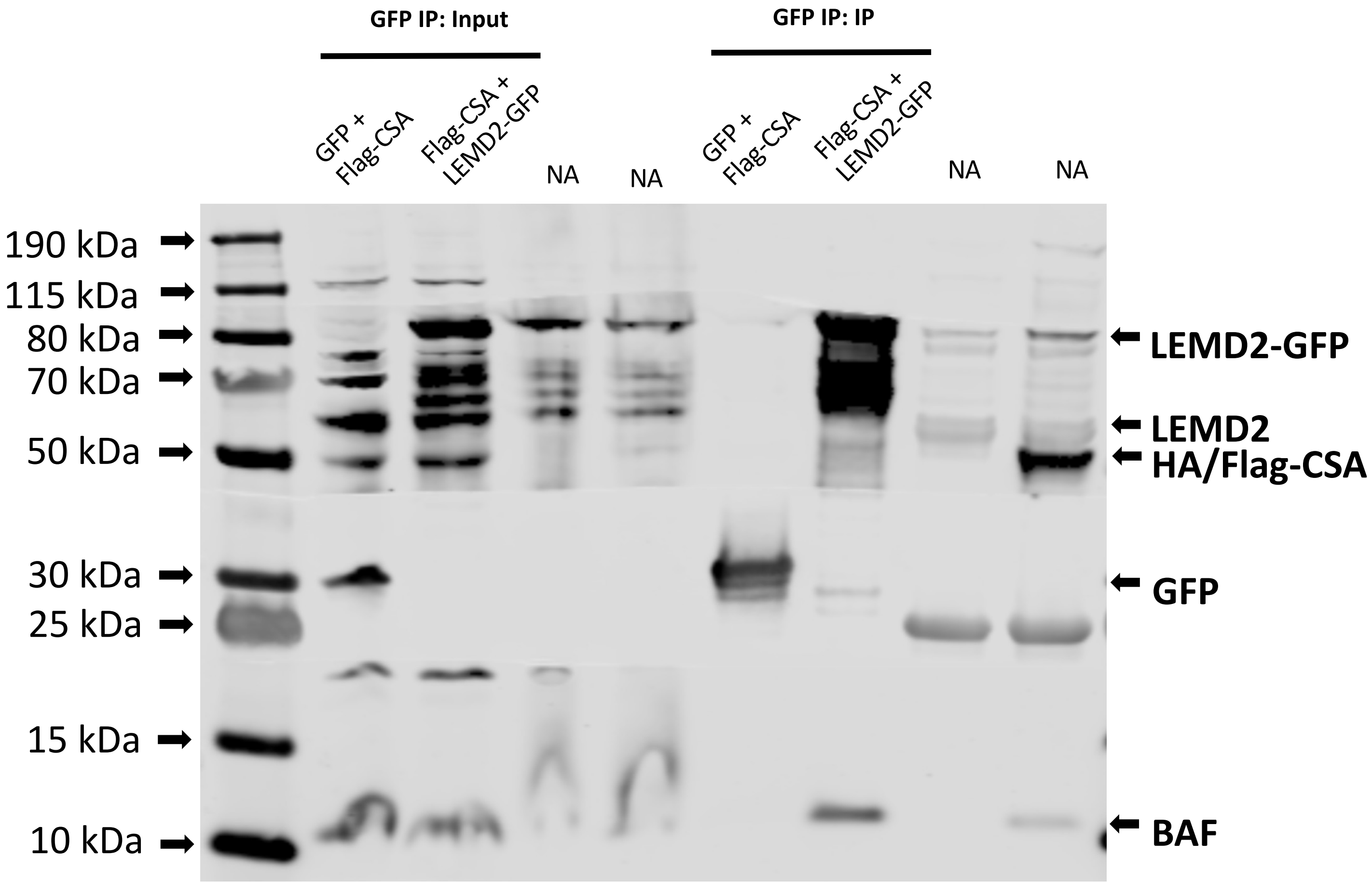
GFP IP: Input
GFP IP: IP
GFP + Flag-CSA
Flag-CSA + LEMD2-GFP
NA
NA
GFP + Flag-CSA
Flag-CSA + LEMD2-GFP
NA
NA
190 kDa
115 kDa
80 kDa
70 kDa
50 kDa
30 kDa
25 kDa
15 kDa
10 kDa
LEMD2-GFP
LEMD2
HA/Flag-CSA
GFP
BAF

Figure 7A

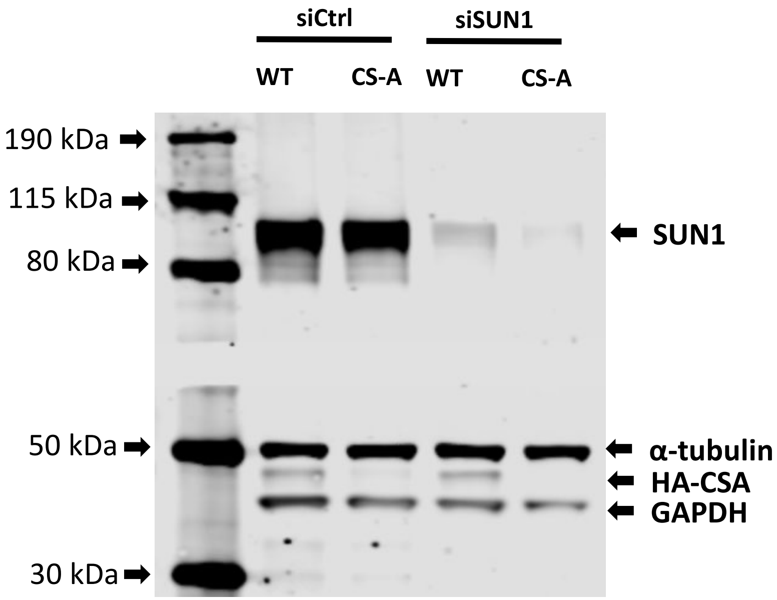
siCtrl
siSUN1
WT
CS-A
WT
CS-A
190 kDa
115 kDa
80 kDa
50 kDa
30 kDa
SUN1
α-tubulin
HA-CSA
GAPDH

Figure 8A

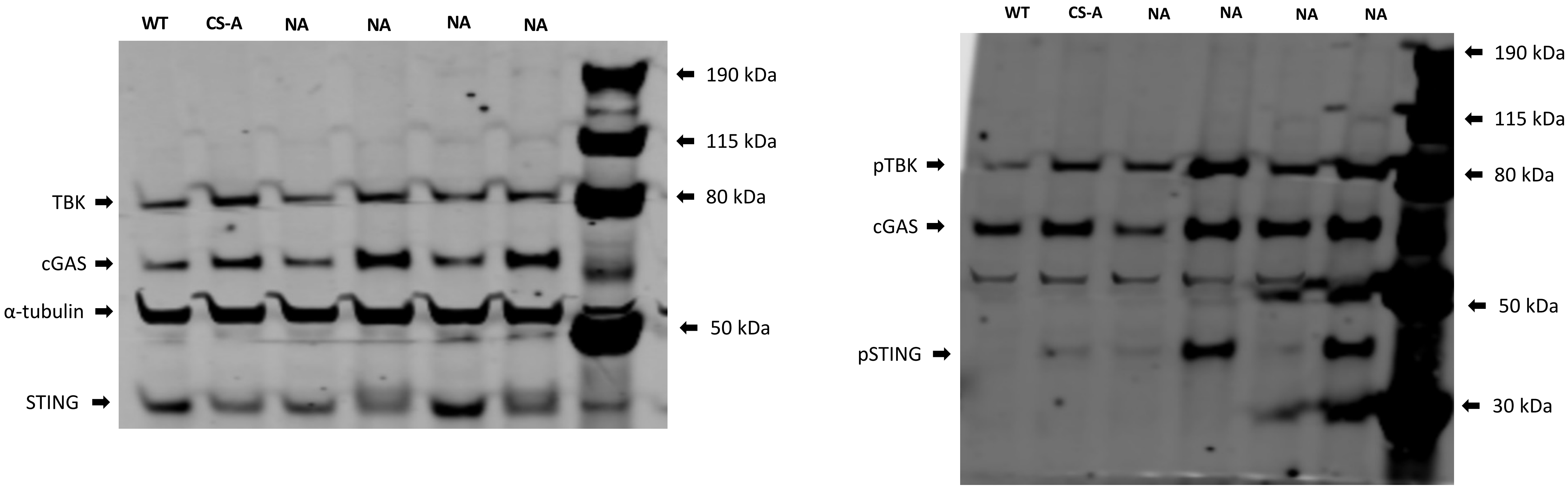
WT
CS-A
NA
NA
NA
NA
190 kDa
115 kDa
80 kDa
50 kDa
TBK
cGAS
α-tubulin
STING
WT
CS-A
NA
NA
NA
NA
190 kDa
115 kDa
80 kDa
50 kDa
30 kDa
pTBK
cGAS
pSTING

Figure 8C

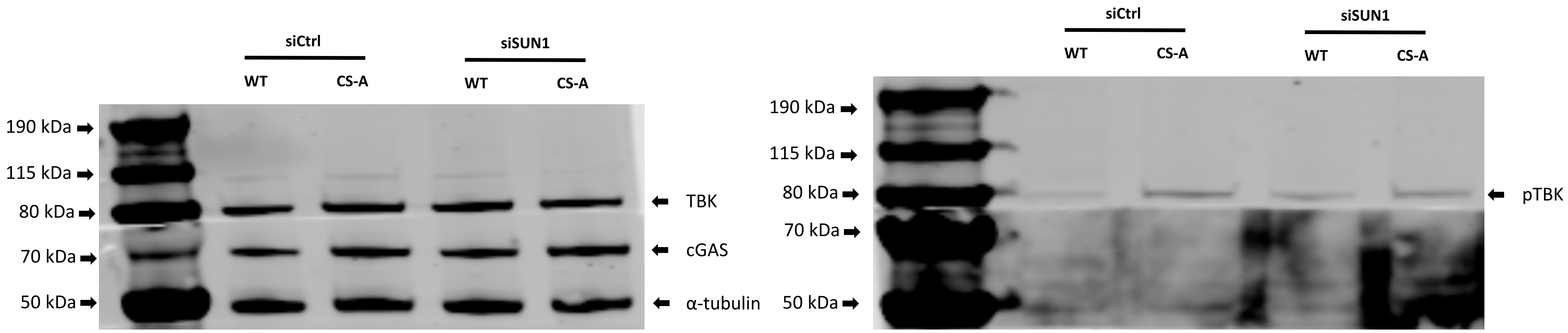
siCtrl
siSUN1
WT
CS-A
WT
CS-A
190 kDa
115 kDa
80 kDa
70 kDa
50 kDa
TBK
cGAS
α-tubulin
siCtrl
siSUN1
WT
CS-A
WT
CS-A
190 kDa
115 kDa
80 kDa
70 kDa
50 kDa
pTBK

Figure 9B

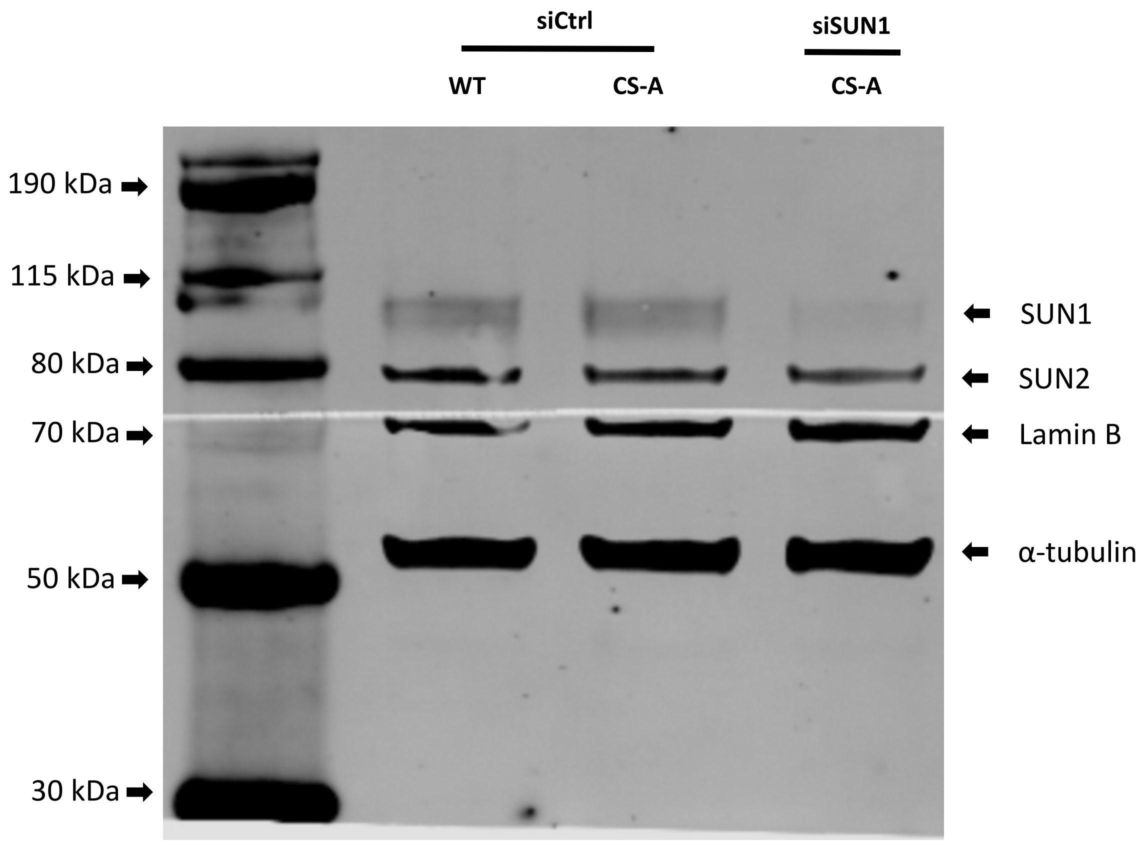
siCtrl
siSUN1
WT
CS-A
CS-A
190 kDa
115 kDa
80 kDa
70 kDa
50 kDa
30 kDa
SUN1
SUN2
Lamin B
α-tubulin

Figure S1A

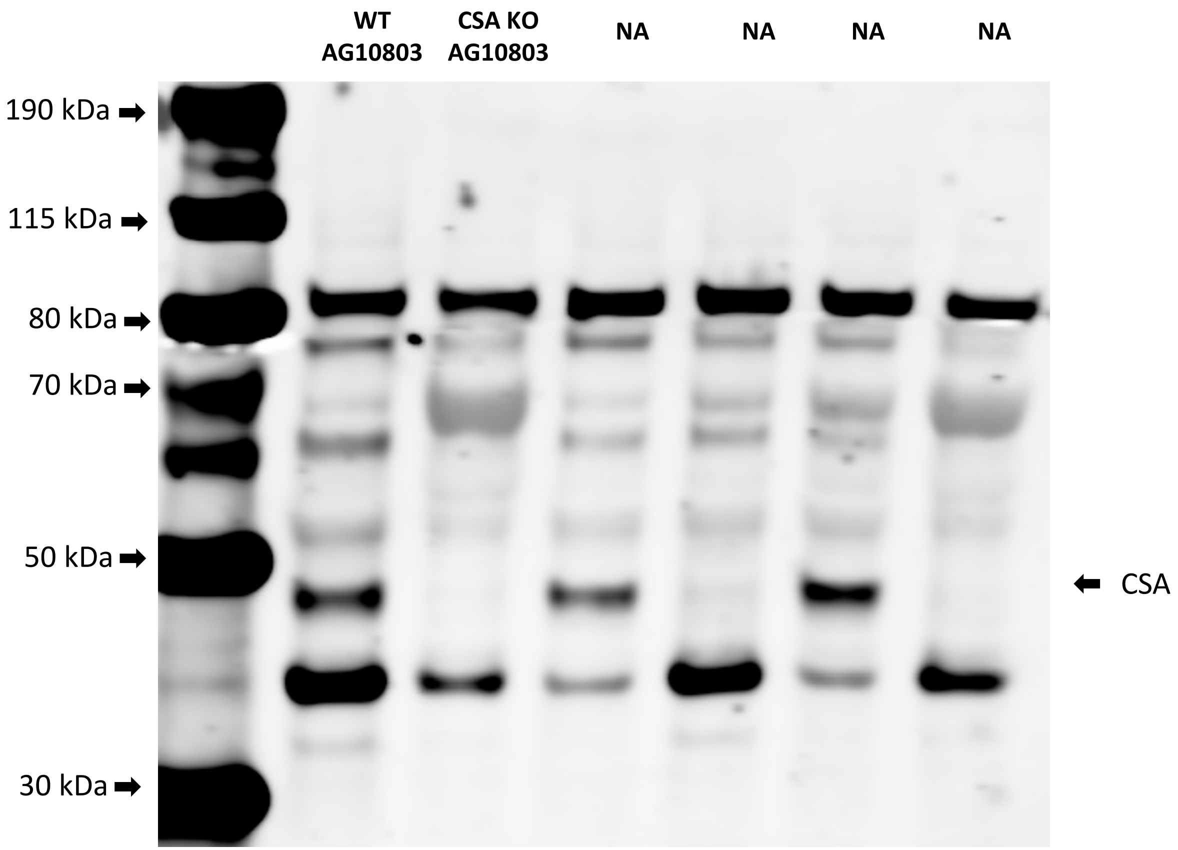
WT AG10803
CSA KO AG10803
NA
NA
NA
NA
190 kDa
115 kDa
80 kDa
70 kDa
50 kDa
30 kDa
CSA

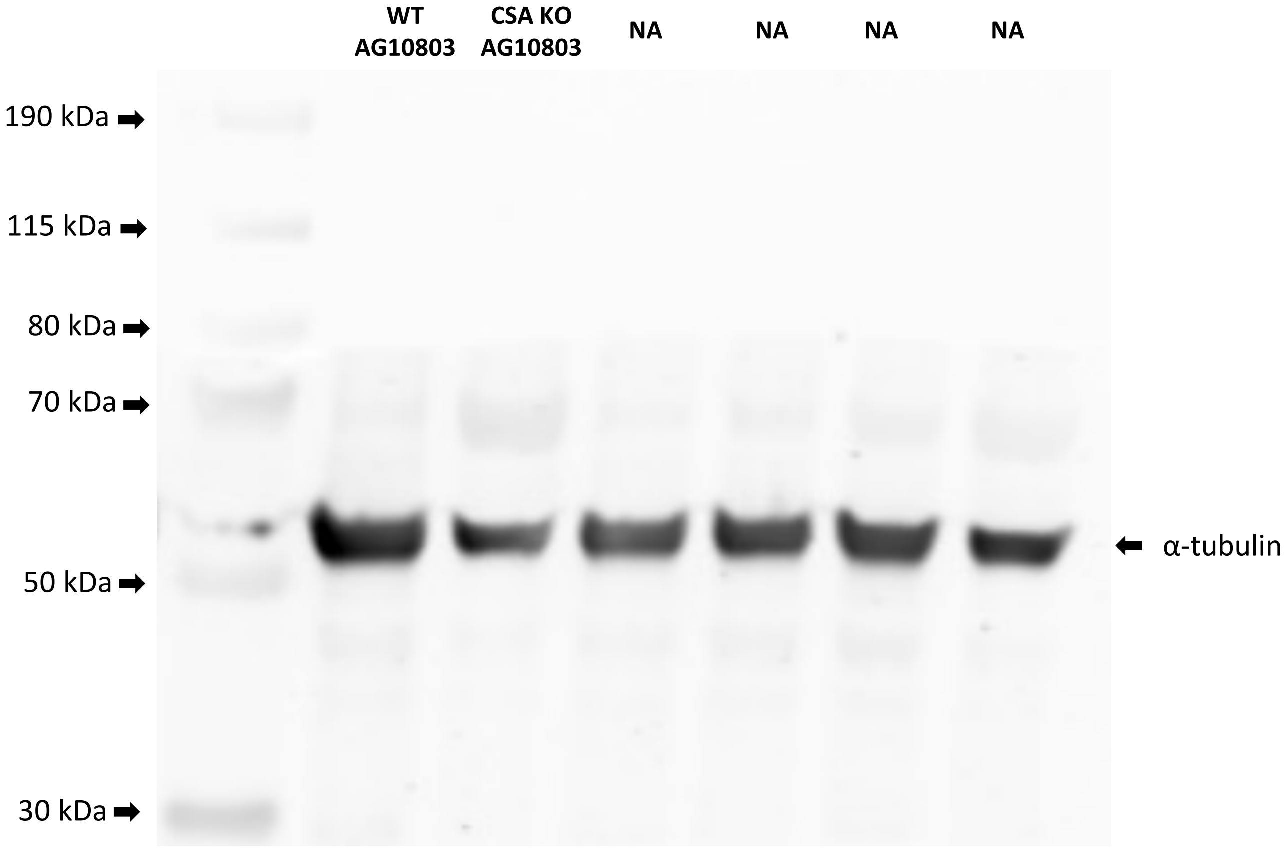
WT AG10803
CSA KO AG10803
NA
NA
NA
NA
190 kDa
115 kDa
80 kDa
70 kDa
50 kDa
30 kDa
α-tubulin

Figure S4A-C

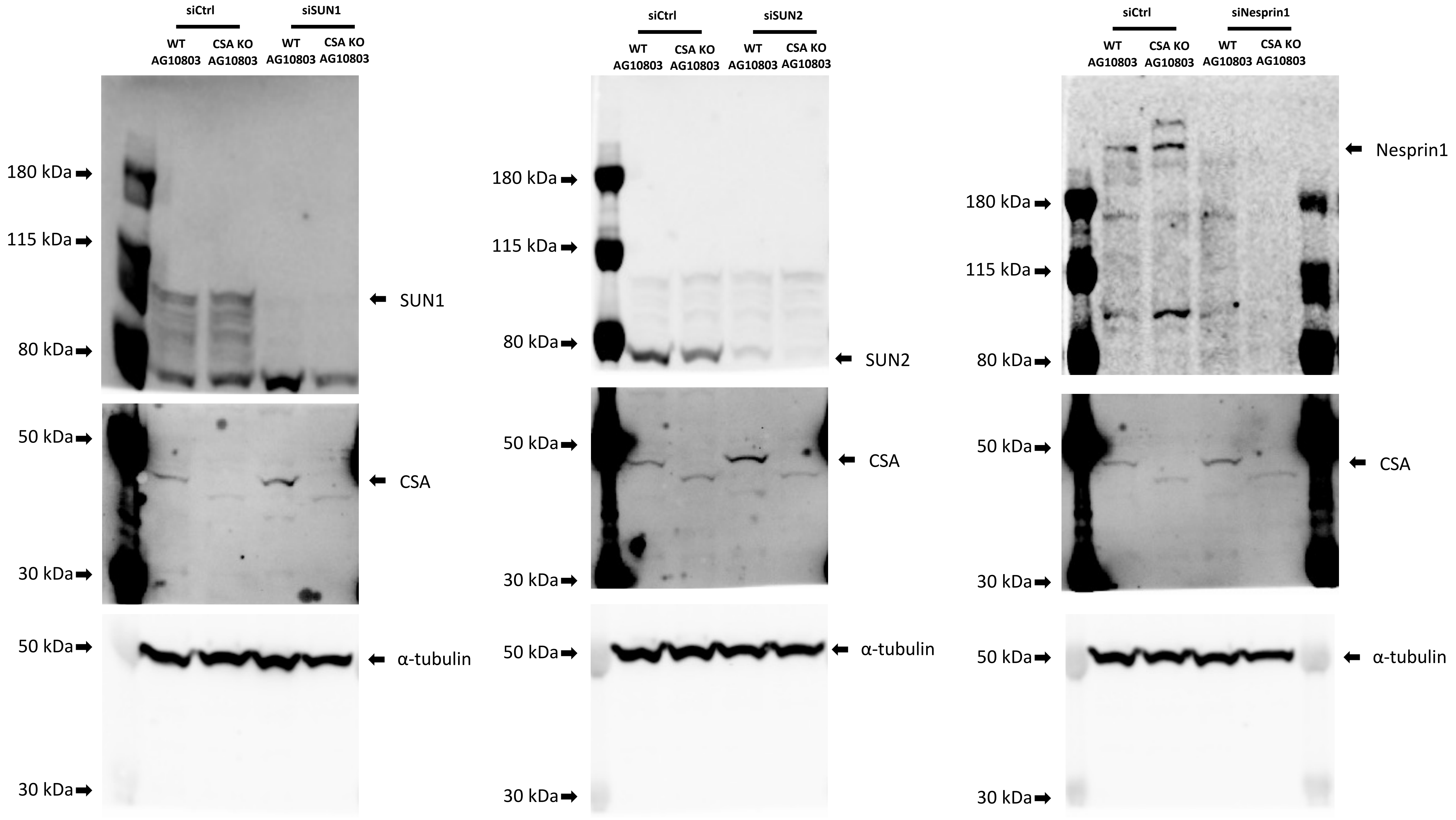
siCtrl
siSUN1
WT AG10803
CSA KO AG10803
WT AG10803
CSA KO AG10803
180 kDa
115 kDa
80 kDa
SUN1
50 kDa
30 kDa
CSA
50 kDa
30 kDa
α-tubulin
siCtrl
siSUN2
WT AG10803
CSA KO AG10803
WT AG10803
CSA KO AG10803
180 kDa
115 kDa
80 kDa
SUN2
50 kDa
30 kDa
CSA
50 kDa
30 kDa
α-tubulin
siCtrl
siNesprin1
WT AG10803
CSA KO AG10803
WT AG10803
CSA KO AG10803
Nesprin1
180 kDa
115 kDa
80 kDa
50 kDa
30 kDa
CSA
50 kDa
30 kDa
α-tubulin

Figure S4D-E

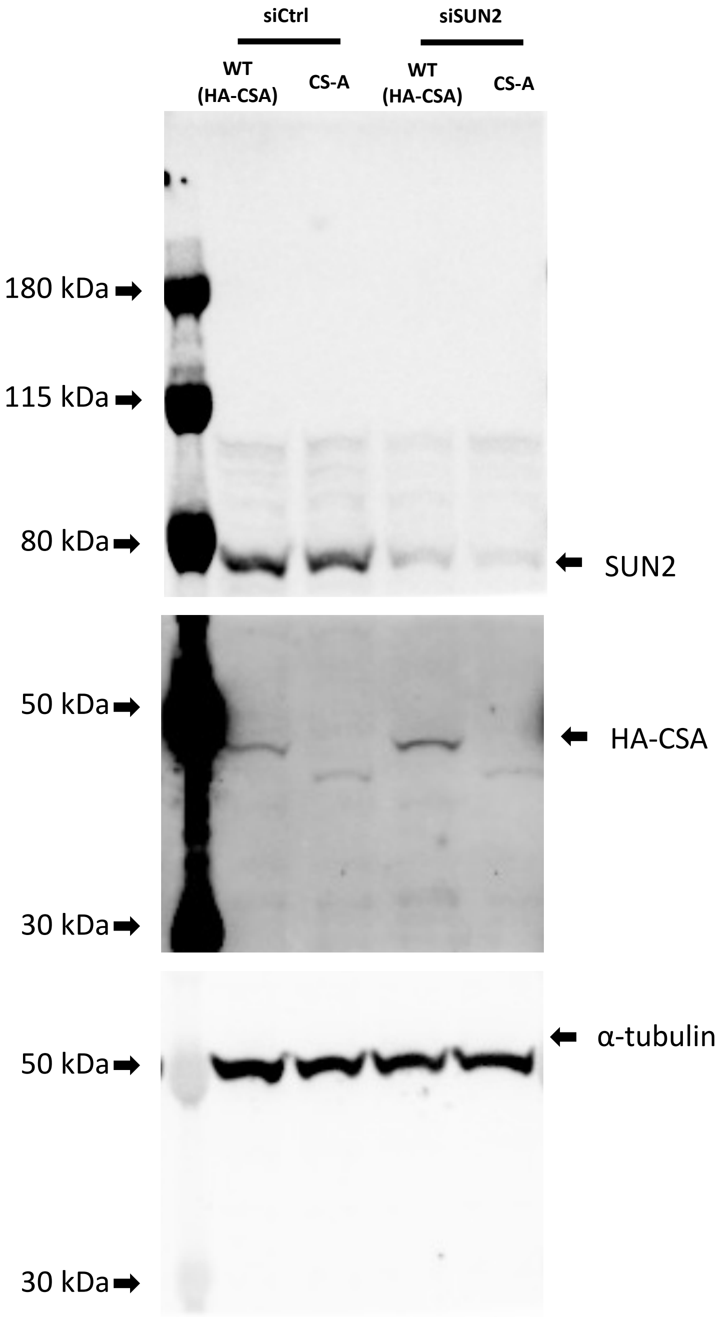
siCtrl
siSUN2
WT (HA-CSA)
CS-A
WT (HA-CSA)
CS-A
180 kDa
115 kDa
80 kDa
SUN2
50 kDa
30 kDa
HA-CSA
α-tubulin
50 kDa
30 kDa

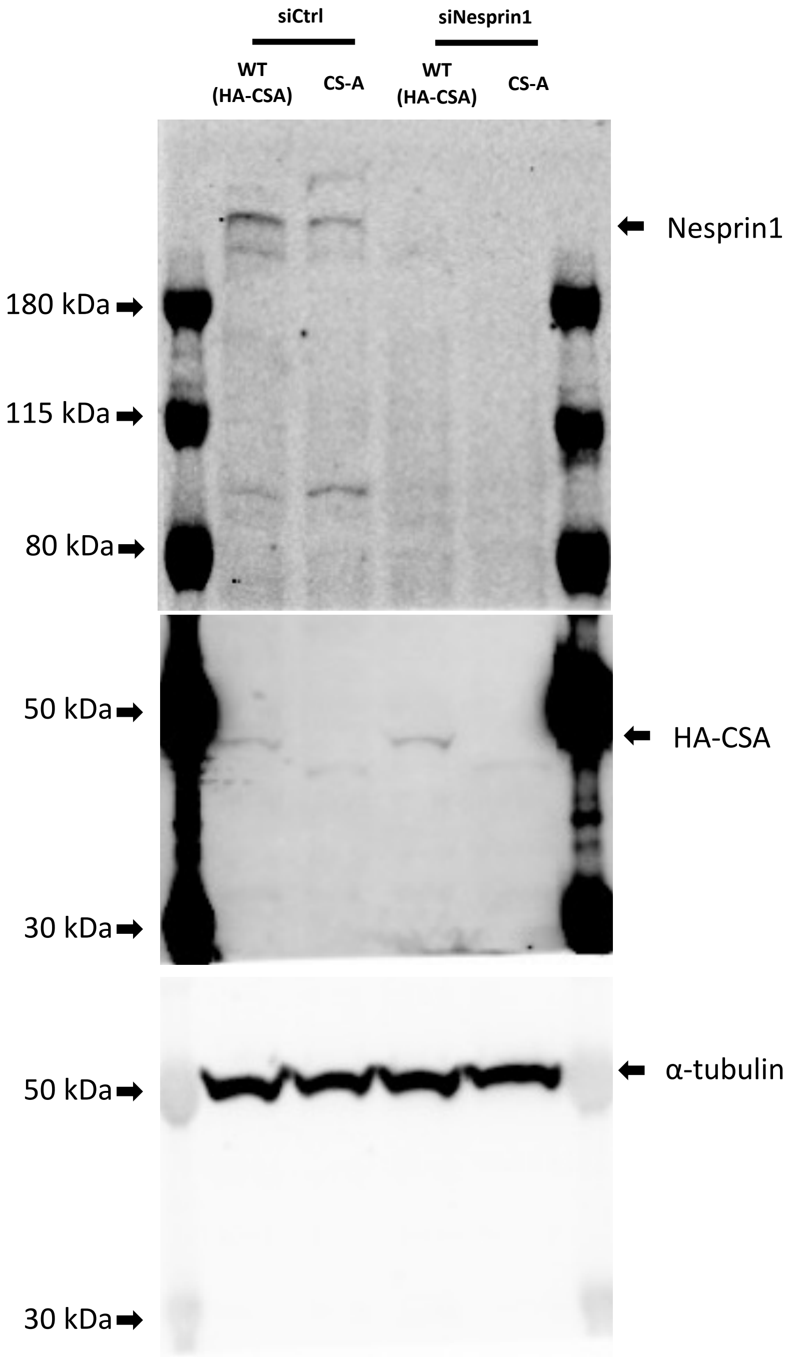
siCtrl
siNesprin1
WT (HA-CSA)
CS-A
WT (HA-CSA)
CS-A
Nesprin1
180 kDa
115 kDa
80 kDa
50 kDa
HA-CSA
30 kDa
α-tubulin
50 kDa
30 kDa

Figure S5A

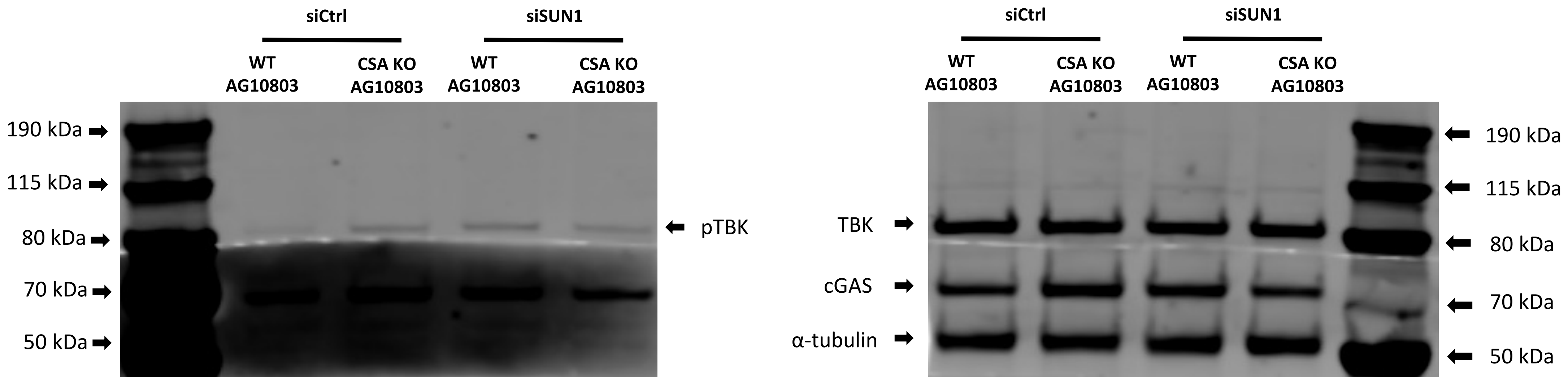
siCtrl
siSUN1
WT AG10803
CSA KO AG10803
WT AG10803
CSA KO AG10803
190 kDa
115 kDa
80 kDa
70 kDa
50 kDa
pTBK
siCtrl
siSUN1
WT AG10803
CSA KO AG10803
WT AG10803
CSA KO AG10803
TBK
cGAS
α-tubulin
190 kDa
115 kDa
80 kDa
70 kDa
50 kDa

Figure S5B

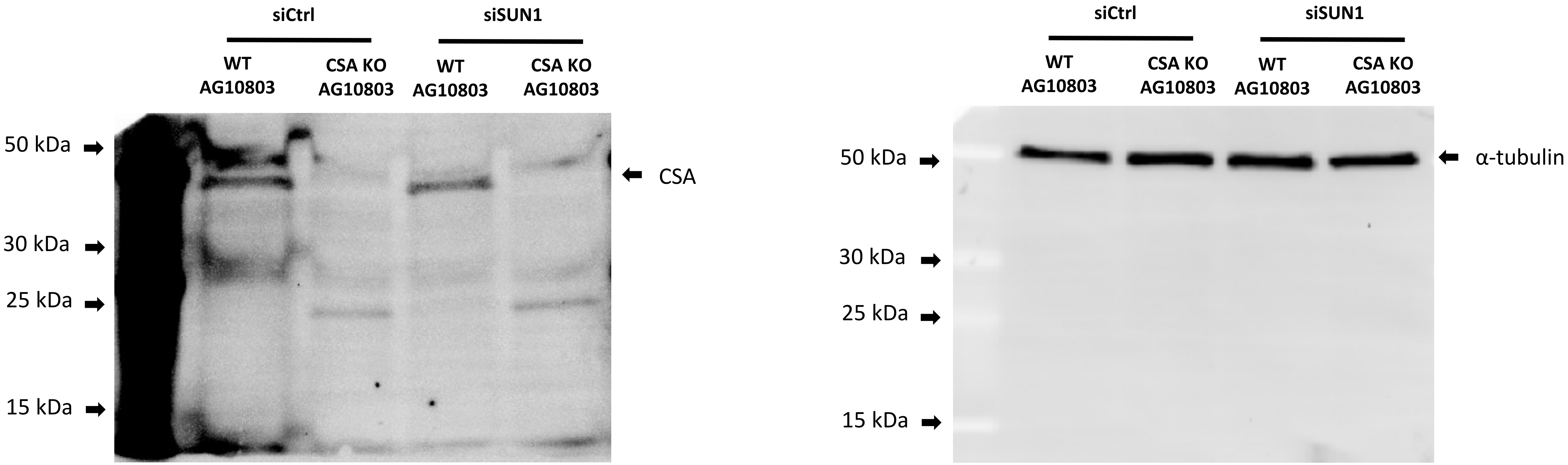
siCtrl
siSUN1
WT AG10803
CSA KO AG10803
WT AG10803
CSA KO AG10803
50 kDa
30 kDa
25 kDa
15 kDa
CSA
siCtrl
siSUN1
WT AG10803
CSA KO AG10803
WT AG10803
CSA KO AG10803
50 kDa
30 kDa
25 kDa
15 kDa
α-tubulin
